# Efficacy and Mechanism of Hypofractionation Radiotherapy Combined with PD-1 Inhibitors in a Model of Head and Neck Melanoma

**DOI:** 10.3390/cancers16030675

**Published:** 2024-02-05

**Authors:** Gaofei Yin, Wei Guo, Xiaohong Chen, Yang Zhang, Zhigang Huang

**Affiliations:** Beijing Tongren Hospital, Capital Medical University, No. 1 Dongjiaomin Lane, Dongcheng District, Beijing 100730, China; qiqi.mail.y@163.com (G.Y.); trchxh@163.com (X.C.); zhangyangent@163.com (Y.Z.)

**Keywords:** head and neck mucosal melanoma, hypofractionation radiotherapy, PD-1 inhibitor, immune humanized tumor model, tumor immune microenvironment

## Abstract

**Simple Summary:**

As a highly malignant and rare tumor, an improvement in treatment efficacy for head and neck mucosal melanoma has always been a key and difficult point in clinical research. We validate the efficacy of hypofractionation radiotherapy combined with PD-1 inhibitor by constructing tumor-humanized and immune-humanized models; confirm that the combination therapy has an impact on T cell immunity; and provide preclinical data reference for subsequent clinical treatment.

**Abstract:**

Head and neck mucosal melanoma is one of the most common types of melanoma in China, but the prognosis is worse than other types, and there is no effective treatment plan to improve patient survival. This study analyzes the efficacy of hypofractionation radiotherapy combined with PD-1 inhibitor in the treatment of head and neck mucosal melanoma, as well as its impact on the tumor immune microenvironment. NPSG mice were used to construct a humanized bilateral lesion tumor model of the humanized immune system. The models were divided into an RT (8 Gy)+anti PD-1 group, an RT (2 GyX4)+anti PD-1 group, an Anti PD-1 group, an RT (8 Gy) group, and a blank group. Differences in efficacy and immune cells in blood, lymph nodes, and tumor tissues were compared between different treatment groups. The treatment effect of RT (8 Gy)+anti PD-1 was better than the other groups with a tumor growth inhibition value (TGI) over 60%. Significant recruitment and activation of CD8+T cells were found in the blood, lymph nodes, and tumor tissues and significantly inhibited the level of PD-1+CD8+T cells in the group of RT (8 Gy)+anti PD-1. This study confirmed the efficacy of hypofractionation radiotherapy combined with PD-1 inhibitors, which can inhibit tumor growth and produce distant effects. The appearance of a distant effect is related to the enhancement in the number and activity of CD8+T cells in the local tumor and peripheral blood and lymph nodes. This study confirms the therapeutic and immune regulatory effect of hypofractionation radiotherapy combined with PD-1 inhibitors.

## 1. Introduction

Head and neck mucosal melanoma (HNMM), as a rare malignant tumor, accounts for only 0.03% of the total tumor incidence, but it is more common in China [1]. There are about 20,000 new cases of melanoma in China every year, and mucosal melanoma accounts for 23%, of which the head and neck incidence rate accounts for about 40% [2]. Due to its highly malignant disease characteristics, the overall 5-year survival rate is only about 20%. Existing adjuvant treatments, including immunotherapy, have not been able to effectively control the disease progression in patients. After undergoing surgery and postoperative adjuvant treatment, the local recurrence and distant metastasis rates can still be as high as 80% [3]. How to effectively control local and distant metastatic lesions in late-stage patients to prolong their survival time has become a key and difficult point in clinical diagnosis and treatment. Immunotherapy has brought new hope for the treatment of advanced melanoma patients [4]. However, the immune checkpoint inhibitor response rate of mucosal melanoma is only 0–12%, and the treatment effect is not ideal [5]. The single use of an immune checkpoint inhibitor is not sufficient to improve the multiple immunosuppressive states of the local immune microenvironment of mucosal melanoma tumors, and seeking more effective combination therapy has become a new direction for tumor immunotherapy. Our previous analysis found that postoperative radiotherapy can effectively improve the local control rate of the disease, and the previous literature also suggests that a single moderate hypofractionation radiotherapy can activate CD8+T cells, followed by a decrease in myeloid suppressor cell levels. However, the specific radiation dose and method still need further exploration. The therapeutic effect of melanoma is still in the exploratory stage. In an animal experiment combining T cell immunotherapy for melanoma, it was confirmed that a single hypofractionation radiotherapy has better efficacy than conventional fractionated radiotherapy [6]. Laboratory studies have also confirmed that large segmentation schemes can induce tumor immunity without damaging local immune cells [7]. Therefore, the aim of this study is to construct an immune-humanized human tumor xenograft (PDX) mouse model, simulate human immunity and tumor status, and explore the therapeutic effect of hypofractionation radiotherapy combined with PD-1 inhibitors, as well as the impact on the local and distant immune status of head and neck mucosal melanoma. We also explore the mechanism of hypofractionation radiotherapy combined with PD-1 inhibitors.

## 2. Material and Methods

### 2.1. Experimental Animals and Tissue Cell Samples

Six- to eight-week-old female NPSG mice were purchased from Shanghai Jihui Experimental Animal Breeding Co., Ltd. The source of human head and neck mucosal melanoma samples was patients in our hospital. The size of the tissue block was approximately 1–0.5 cm^3^. After the sample was detached, it was placed in tissue preservation solution and transported to the laboratory at 4 °C for insulation. Mouse tumor tissue source: After processing, human tumor tissue samples were inoculated into immunodeficient mice and passaged to 2–3 generations to avoid enhanced tumor heterogeneity. The tumor blocks were frozen and stored for future use. Human PBMC was tested using PBMC donors screened in vitro. Supplier: Miaosun (Shanghai) Biotechnology Co., Ltd. (Shanghai, China) Specification: 1 × 10^8^/vial. Donor ID: # 701C. The collection of experimental mice and tissue samples was approved by the Ethics Committee of Beijing Tongren Hospital Affiliated with Capital Medical University.

### 2.2. The PDX Model Construction Method for Immune Reconstruction

Forty 6–8-week-old female NPSG mice were fed adaptively for 1 week and weighed. Tumor tissue was collected from tumor xenograft model-bearing mice, and the tumor tissue was digested into single cells and counted for later use. We chose mice with uniformly sized tumors, so 10 mice did not participate in the experiment. Inoculation method: Mouse tumor cells were inoculated subcutaneously on both sides of the back of NPSG mice (subcutaneously on the left and right shoulders), with each side containing 5 × 10^5^ tumor cells. One side was the experimental side, and the other side was the control side. PBMC was then resuscitated, counted, and inspected for backup. After one week of inoculating the tumor cells, all grouped mice were inoculated with PBMC through the tail vein.

### 2.3. Treatment Plan and Grouping

When the average tumor volume reached about 80–150 mm^3^, the mice were randomly divided into 5 groups with 6 mice in each group (Table 1). The grouping date was considered day 0. RT (8 Gy)+anti PD-1 group: a single dose of 8 Gy was used to irradiate one side (intervention side, right tumor single lesion irradiation) of the tumor area once, with a PD-1 inhibitor treatment of 10 mg/kg for 2–3 weeks, twice a week (BIW). RT (2 GyX4)+anti PD-1 group: 2 Gy single-dose irradiation on one side (intervention side, right tumor single lesion irradiation) of the tumor area 4 times, during which a PD-1 inhibitor treatment of 10 mg/kg was administered for 2–3 weeks, twice a week (BIW). Anti PD-1: received only 2–3 weeks of PD-1 inhibitor treatment of 10 mg/kg twice a week (BIW); the remaining radiotherapy time was released together with other groups of mice. RT (8 Gy) group: only received a single dose of 8 Gy irradiation on one side (intervention side, right tumor single lesion irradiation) of the tumor area once; an equal dose of physiological saline was injected at the same time as the PD-1 inhibitor group. Blank group: the mice in the Blank group were released together with the other groups but without irradiation, and they were injected with the same dose of physiological saline at the same time as the other groups. The weight of the experimental animals was measured once a week from vaccination to grouping. After grouping, the weight of the experimental animals was measured twice a week (combined with treatment time). The tumor volume of the experimental animals was measured once a week. After vaccination and grouping, the bilateral tumor volume of the experimental animals was measured twice a week (combined with treatment time). The tumor volume measurement followed a bidirectional measurement method, first measuring the length and diameter of the tumor using a vernier caliper and then using the formula TV = 0.5 × *a* × *b*^2^, where *a* is the long diameter of the tumor and *b* is the short diameter of the tumor.

### 2.4. Flow Cytometry Analysis

Tumor cell suspensions were stained, and single-cell analysis was performed by flow cytometry using 8-parameter flow cytometry on a FACS Canto (BD). To identify positive and negative populations, gates were set based on pilot experiments and our experience running similar experiments. Fluorescence minus one, isotype controls, and/or negative samples were used to determine gate boundaries. Additionally, antibody titration was used to optimize antibody panels. Immune phenotyping of samples was purchased from Biolegend (FVD eFluor 660, hCD3-Alexa Fluor 700, hCD8 PerCP-Cy5.5, mCD45 PE-CY7, hCD45 APC-eFluor 780, hCD279 FITC, Granzyme B PE, hCD4 FITC, hCD25 PE-Cy7, Foxp3 PE, hFOXP3 PE, hIFN-γ PerCP-Cy5.5).

### 2.5. Immunofluorescence

Wax was removed from the tumor tissue section (prepared and embedded with the assistance of our pathology department) and poured into water. This was followed by antigen microwave repair, at a temperature of 92–96 °C for 10–15 min, and natural cooling to room temperature. Normal sheep serum was blocked at 37 °C for 60 min. The excess serum was poured out, and the first antibody was added dropwise overnight at 37 °C for 2 h or 4 °C, followed by rinsing with PBS for 5 min × 3 times. The fluorescein-labeled secondary antibody was then dripped, avoiding light, and rinsed at 37 °C, for 60 min, in 0.01 mL PBS, 5 min × 3 times each. It was sealed with an anti-quenching sealing agent and stored at 4 °C in a dark place. We used 3D HISTECH to scan slices and adjust and save them in the Case Viewer.

### 2.6. Statistical Methods

For pairwise comparisons, the T-test analysis method was used. For potential synergistic effects, a two-way ANOVA was used. All data analyses were conducted with SPSS 24.0. A *p*-value less than 0.05 was considered a significant difference. We applied Graph Prism 9.0 to plot tumor growth curves and mouse body weight curves.

## 3. Results

### 3.1. Combination Radiation and Anti-PD-1 Therapy Induces a Systemic Anti-Tumor Response

#### 3.1.1. Changes in Body Weight of Mice in Each Group

**Figure 1:**
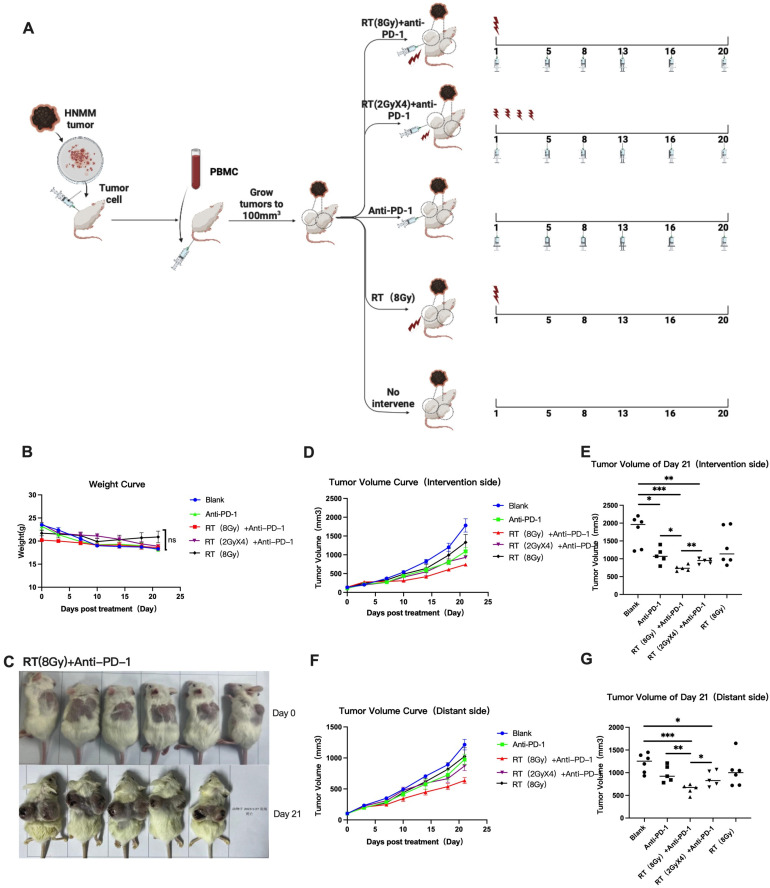
(**A**) Treatment grouping and treatment plan of this study. (**B**) The growth curve of mice. ns: *p* = 0.1624. (**C**) Animal model photo of the RT (8 Gy)+anti PD-1 group. Chinese text: One mouse died on 27 January 2023. (**D**) Tumor volume curve of intervention sides. (**E**) Tumor volume of Day 21 in each groups of intervention sides. * *p* = 0.0108; *p* = 0.0110; ** *p* = 0.0020; *p* = 0.0046; *** *p* = 0.0005. (**F**) Tumor volume curve of distant sides. (**G**) Tumor volume curve of distant sides. * *p* = 0.0166; *p* = 0.0313; ** *p* = 0.0075; *** *p* = 0.0003.

After screening for tumor volume uniformity, the body weight of each group of mice during the experimental treatment cycle was recorded. The weight change curve of each group of mice is shown in Figure 1, indicating a fluctuation of ±5 g in weight. As of the end of the experiment, there was no statistically significant difference in weight changes among the mice in each group.

#### 3.1.2. Changes in Tumor Size in Each Group

During the process from the beginning of treatment to the end of the experiment, five groups of mice were used to measure the length and diameter of the tumor using a vernier caliper and to calculate the tumor volume. The tumor volume of each group was recorded and calculated on the 0th, 3rd, 7th, 10th, 14th, 18th, and 21st days after the start of treatment, and the average ± standard error of the tumor size was calculated for recording. The tumor growth curve was plotted, as shown in Figure 1. At the end of the experiment, compared with the Blank group, the RT (8 Gy) group, Anti PD-1 group, RT (2 GyX4)+anti PD-1 group, and RT (8 Gy)+anti PD-1 group all showed a certain tumor inhibition effect (*p* = 0.1326; *p* = 0.0108; *p* = 0.0020; *p* = 0.0005). The RT (8 Gy)+anti PD-1 group showed the most significant effect, and the tumor inhibition rate of the RT (8 Gy)+anti PD-1 group was better than that of the Anti PD-1 group and the RT (2 GyX4)+anti PD-1 group (*p* = 0.0110, *p* = 0.0046), while there was no difference in the tumor inhibition rate between the Anti PD-1 group and the RT (2 GyX4)+anti PD-1 group (*p* = 0.1687). The same results were reflected in the size of distant tumors. Compared with the control group, the RT (8 Gy) group, Anti PD-1 group, RT (2 GyX4)+anti PD-1 group, and RT (8 Gy)+anti PD-1 group all showed a certain tumor inhibition effect (*p* = 0.2735; *p* = 0.0787; *p* = 0.0166; *p* = 0.0003). The RT (8 Gy)+anti PD-1 group showed the most significant effect, and the tumor inhibition rate of the RT (8 Gy)+anti PD-1 group was better than that of the Anti PD-1 group and the RT (2 GyX4)+anti PD-1 group (*p* = 0.0075, *p* = 0.0313), while there was no difference in the tumor inhibition rate between the Anti PD-1 group and the RT (2 GyX4)+anti PD-1 group (*p* = 0.3808).

The results of calculating the tumor growth inhibition rates on both sides of the mice at the end of each experiment are shown in Table 2. The TGI value of the RT (8 Gy)+anti PD-1 group was 63.5%, which was greater than 60%, thus indicating a strong pharmacological effect. The TGI value of the RT (2 GyX4)+anti PD-1 group was 51.2%, which was less than 60% but greater than 30%, thus indicating moderate efficacy. The TGI value of the Anti PD-1 group was 40.4%, which was less than 60% but greater than 30%, thus indicating moderate efficacy. The TGI value of the RT (8 Gy) group was 26.7%, which was less than 30%, thus indicating no efficacy. It can be seen that RT (8 Gy)+anti PD-1 can achieve a relatively ideal tumor inhibition rate.

The results of the tumor growth inhibition rate on the distant side are shown in Table 3. The TGI value of the RT (8 Gy)+anti PD-1 group was 52.0%. The TGI value of the RT (2 GyX4)+anti PD-1 group was 30.9%, which was less than 60% but greater than 30%, thus indicating moderate efficacy. The TGI value of the Anti PD-1 group was 21.4% and the TGI value of the RT (8 Gy) group was 16.9%, which were both less than 30% and can be considered ineffective. RT (8 Gy)+anti PD-1 can also achieve an ideal tumor inhibition rate for distant lesions.

### 3.2. Irradiation and Anti-PD-1 Therapy Enhance Anti-Tumor T Cell Immunity

#### 3.2.1. Level and Function of T Cells in Peripheral Blood

Blood samples from the inner canthus vein of mice were taken on day 0 of the experiment, before each cycle of medication (day 7, day 15), and at the end of the experiment (day 21). The proportion of various cells and their changes during the experimental cycle were analyzed by flow cytometry, including effector T cells, memory T cells, initial T cells, CD8+T cells, and PD-1+CD8+T cells.

The proportion changes of CD8+T cells and PD-1+CD8+T cells in peripheral blood were plotted on the 0th, 7th, 15th, and 21st days of the experiment as a cellular change level curve and then statistical analyses were conducted.

**Figure 2:**
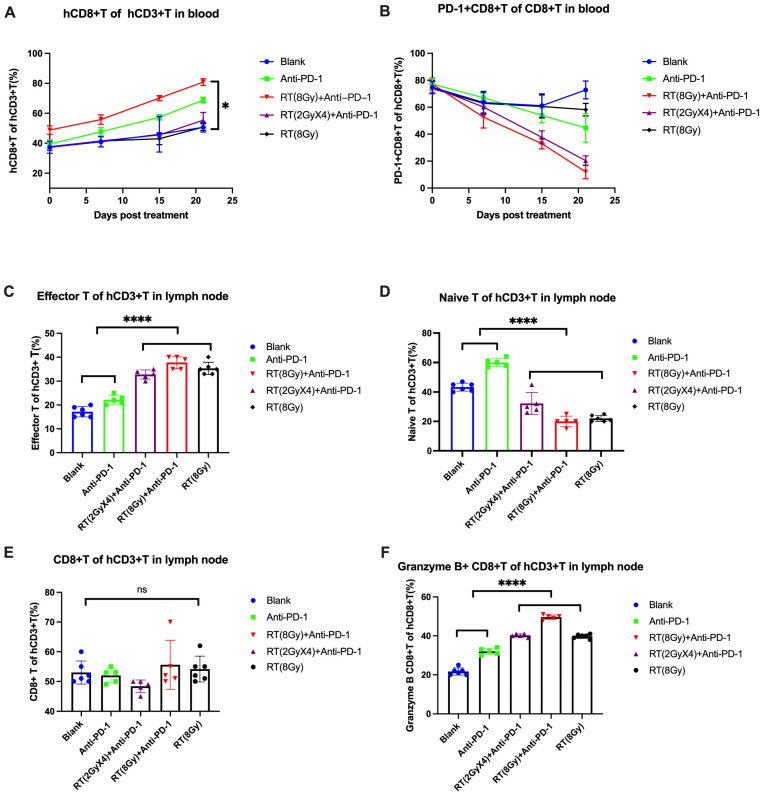
(**A**,**B**) CD8+T cells in the blood of different groups. (**C**–**F**) Effector T cells, naive T cells, CD8+T cells, and granzyme B+T cells in the lymph nodes of different groups.**** *p* < 0.0001; * *p* = 0.040; ns: *p* = 0.0955.

From the cell change curve, the CD8+T cells in each group showed an overall upward trend throughout the entire experimental cycle. Intergroup differences were analyzed (Figure 2A), and compared with the Blank control group, the CD8+T cell levels in the RT (8 Gy)+anti PD-1 group showed statistical differences (*p* = 0.0404). From the cell change curve, the PD-1+CD8+T cells in the Blank control group showed a stable overall trend with a slight increase throughout the experimental cycle, while the levels of PD-1+CD8+T cells in the other treatment groups showed a downward trend (Figure 2B), but the differences between the groups did not have a continuous significance.

#### 3.2.2. Level and Function of T Cells in Lymph Nodes

At the end of the experiment, the inguinal lymph node tissue of mice was collected and detected by flow cytometry to analyze the proportion of effector T cells and initial T cells in the lymph node tissue. For effector T cells, compared with the Blank group, the RT (8 Gy) group, the Anti PD-1 group, the RT (2 GyX4)+anti PD-1 group, and the RT (8 Gy)+anti PD-1 group all showed a certain level of high expression (*p* < 0.0001; *p* < 0.0001; *p* = 0.0028; *p* < 0.0001), and the RT (8 Gy)+anti PD-1 group showed the most significant expression. There were significant differences in effector T cell levels compared with the Anti PD-1 group and the RT (2 GyX4)+anti PD-1 group (*p* < 0.0001; *p* = 0.0085). The expression of effector T cells in the treatment group with increased radiation therapy was higher than that in the Anti PD-1 group (*p* < 0.0001). For the initial T cells, compared with the Blank group, the RT (8 Gy) group, the Anti PD-1 group, the RT (2 GyX4)+anti PD-1 group, and the RT (8 Gy)+anti PD-1 group all showed a certain level of low expression (*p* < 0.0001; *p* < 0.0001; *p* = 0.0078; *p* < 0.0001), and the RT (8 Gy)+anti PD-1 group showed the most significant low expression. Compared with the initial T cell levels of the Anti PD-1 group, RT (2 GyX4)+anti PD-1 group, there were significant differences (*p* < 0.0001; *p* = 0.0110), and the initial T cell expression in the treatment group with increased radiation therapy was lower than that in the Anti PD-1 group (*p* < 0.0001) (Figure 2C,D).

In addition, the statistical analysis of CD8+T cell levels found that there was no statistically significant difference between the treatment groups. We analyzed the levels of Granzyme B+CD8+T cells (killer T cells) and explored the differences in levels of cytotoxic T cells. Compared with the Blank group, the RT (8 Gy) group, the Anti PD-1 group, the RT (2 GyX4)+anti PD-1 group, and the RT (8 Gy)+anti PD-1 group all showed a certain level of high expression (*p* < 0.0001; *p* < 0.0001; *p* < 0.0001; *p* < 0.0001), and the RT (8 Gy)+anti PD-1 group showed the most significant expression. Compared with the Granzyme B+CD8+T cell levels in the Anti PD-1 group and RT (2 GyX4)+anti PD-1 group, there were significant differences (*p* < 0.0001; *p* < 0.0001). The treatment group with increased radiation therapy showed higher levels of Granzyme B+CD8+T cells compared with the Anti PD-1 group (*p* < 0.0001). Radiation therapy can promote immune activation in the lymph nodes of mice, so the effect of T in the lymph nodes increases after radiation therapy. There is no difference in CD8+T, but CD8+Granzyme B+T increases (Figure 2E,F).

#### 3.2.3. Changes in T Cell Levels and Functions in Tumor Tissue

##### Local Infiltration of Functional T-Cell Tumors

**Figure 3:**
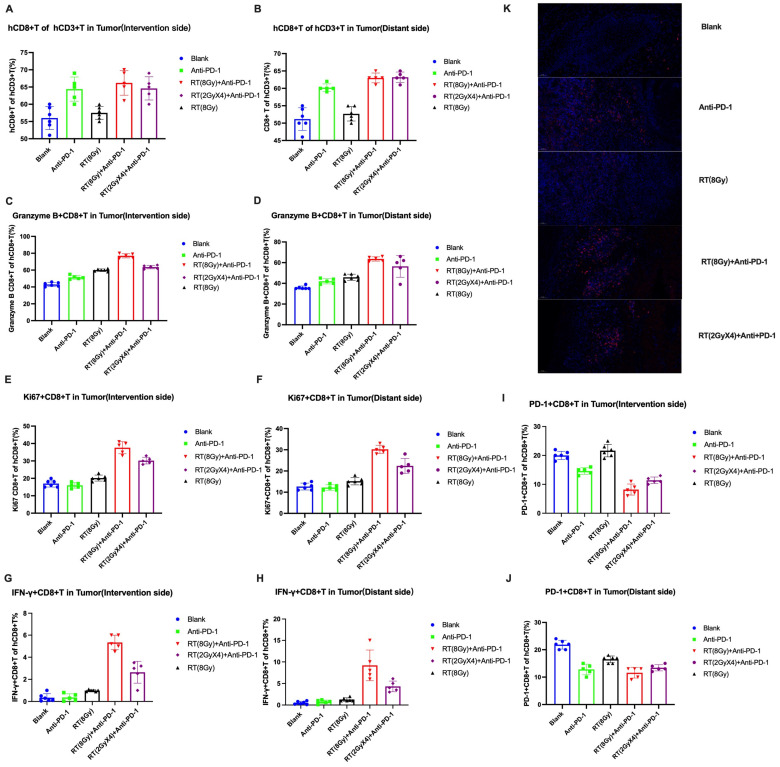
Different types of CD8+T cells on intervention and distant sides. (**A**) CD8+T cell in tumor; (**B**) CD8+T cell in distant tumor; (**C**) Granzyme B+CD8+T cell in tumor; (**D**) Granzyme B+CD8+Tcell in distant tumor; (**E**) Ki67+CD8+T cell in tumor; (**F**) Ki67+CD8+T cell in distant tumor; (**G**) IFN-γ+CD8+Tcell in tumor; (**H**) IFN-γ+CD8+T cells in distant tumor; (**I**) PD-1+CD8+T cell in tumor; (**J**) PD-1+CD8+T cell in distant tumor; (**K**) Differential analysis of CD8+T cells under microscope (scale bar: 50 μm).

We collected tumor tissue from the intervention and distant sides and performed flow cytometry analysis on CD8+T cells and various subtypes (Ki67+CD8+T, IFN-γ+CD8+T, PD-1+CD8+T, and Granzyme B+CD8+T cells (Figure 3).

In the intervention side tumor, the statistical analysis of CD8+T cell levels showed that compared with the Blank group, the Anti PD-1 group, the RT (2 GyX4)+anti PD-1 group, and the RT (8 Gy)+anti PD-1 group all showed a certain level of high expression (*p* = 0.0029; *p* = 0.0022; *p* = 0.0009), and there was no difference between the three treatment groups.

We analyzed the levels of Granzyme B+CD8+T cells (killer T cells) and explored the differences in the levels of cytotoxic T cells. Compared with the Blank group, the RT (8 Gy) group, the Anti PD-1 group, the RT (2 GyX4)+anti PD-1 group, and the RT (8 Gy)+anti PD-1 group all showed a certain level of high expression (*p* = 0.0002; *p* < 0.0001; *p* < 0.0001; *p* < 0.0001), and the RT (8 Gy)+anti PD-1 group showed the most significant expression. Compared with the Granzyme B+CD8+T cell levels in the Anti PD-1 group and the RT (2 GyX4)+anti PD-1 group, there were significant differences (*p* < 0.0001; *p* < 0.0001).

The analysis of the levels of Ki67+CD8+T cells (proliferative T cells) showed that compared with the Blank group, the RT (8 Gy) group, the RT (2 GyX4)+anti PD-1 group, and the RT (8 Gy)+anti PD-1 group all showed a certain level of high expression (*p* = 0.0148; *p* < 0.0001; *p* < 0.0001), and the RT (8 Gy)+anti PD-1 group showed the most significant expression. Compared with the Ki67+CD8+T cell levels in the RT (8 Gy) group and the RT (2 GyX4)+anti PD-1 group, there were significant differences (*p* < 0.0001; *p* = 0.0030). The levels of Ki67+CD8+T cells in the RT (8 Gy) group, the RT (2 GyX4)+anti PD-1 group, and the RT (8 Gy)+anti PD-1 group with increased radiation therapy were all higher than those in the Anti PD-1 group (*p* = 0.0025; *p* < 0.0001; *p* < 0.0001).

The analysis of PD-1+CD8+T cell levels showed that compared with the Blank group, the Anti PD-1 group, the RT (2 GyX4)+anti PD-1 group, and the RT (8 Gy)+anti PD-1 group all showed a certain level of low expression (*p* < 0.0001; *p* < 0.0001; *p* < 0.0001). The RT (8 Gy)+anti PD-1 group showed the most significant low expression, with a significant difference (*p* = 0.0002) compared with the Anti PD-1 group’s PD-1+CD8+T cell levels.

The analysis of IFN-γ+CD8+T cells (secretory T cells) levels showed that compared with the Blank group, the RT (8 Gy) group, the RT (2 GyX4)+anti PD-1 group, and the RT (8 Gy)+anti PD-1 group all showed a certain level of high expression (*p* = 0.0018; *p* < 0.0001; *p* = 0.0005), and the RT (8 Gy)+anti PD-1 group showed the most significant high expression. Compared to the RT (8 Gy) group and the RT (2 GyX4)+anti PD-1 group, the level of IFN- γ+ CD8+T cells showed significant differences (*p* < 0.0001; *p* = 0.0009), indicating a significant immune regulatory effect after increased radiation therapy, activating the local release of IFN- γ in the tumor. The aggregation of secreted CD8+T cells (Tc1 cells) further regulates the host’s immune response.

The above findings were also found in distant tumors. The statistical analysis of CD8+T cell levels showed that compared with the Blank group, the Anti PD-1 group, the RT (2 GyX4)+anti PD-1 group, and the RT (8 Gy)+anti PD-1 group all showed a certain level of high expression (*p* = 0.0002; *p* < 0.0001; *p* < 0.0001). The RT (8 Gy)+anti PD-1 group showed the most significant expression, with a significant difference in CD8+T cell levels compared to the Anti PD-1 group (*p* = 0.0081).

We analyzed the levels of Granzyme B+CD8+T cells (killer T cells) and explored the differences in levels of cytotoxic T cells. Compared with the Blank group, the Anti PD-1 group, the RT (8 Gy) group, the Anti PD-1 group, the RT (2 GyX4)+anti PD-1 group, and the RT (8 Gy)+anti PD-1 group all showed a certain level of high expression (*p* = 0.0008; *p* < 0.0001; *p* = 0.0010; *p* < 0.0001), and the RT (8 Gy)+anti PD-1 group and the RT (2 GyX4)+anti PD-1 group showed significant expression. There was a significant difference in the Granzyme B+CD8+T cell level compared with the Anti PD-1 group (*p* < 0.0001; *p* < 0.0001).

The analysis of the levels of Ki67+CD8+T cells (proliferative T cells) showed that compared with the Blank group, the RT (8 Gy) group, the RT (2 GyX4)+anti PD-1 group, and the RT (8 Gy)+anti PD-1 group all showed a certain level of high expression (*p* = 0.0274; *p* = 0.0002; *p* < 0.0001), and the RT (8 Gy)+anti PD-1 group showed the most significant expression. Compared with the Ki67+CD8+T cell levels in the RT (8 Gy) group and RT (2 GyX4)+anti PD-1 group, there were significant differences (*p* < 0.0001; *p* = 0.0024).

The analysis of PD-1+CD8+T cell levels showed that compared with the Blank group, the RT (8 Gy) group, the Anti PD-1 group, the RT (2 GyX4)+anti PD-1 group, and the RT (8 Gy)+anti PD-1 group all showed a certain level of low expression (*p* < 0.0001; *p* < 0.0001; *p* < 0.0001; *p* < 0.0001). The RT (8 Gy)+anti PD-1 group showed relatively significant low expression, but the difference was not statistically significant compared with the Anti PD-1 group and PD-1+CD8+T group.

The analysis of IFN- γ+ CD8+T cells (secretory T cells) levels showed that compared with the Blank group, the RT (8 Gy) group, the RT (2 GyX4)+anti PD-1 group, and the RT (8 Gy)+anti PD-1 group all showed a certain level of high expression (*p* = 0.0061; *p* < 0.0001; *p* = 0.0002), and the RT (8 Gy)+anti PD-1 group showed the most significant high expression. Compared to the RT (8 Gy) group and RT (2 GyX4)+anti PD-1 group, there was a significant difference in the level of IFN- γ+CD8+T cells (*p* = 0.0008; *p* = 0.0201). The combination of radiotherapy and anti PD-1 also showed significant immune regulatory effects in distant lesions.

The results showed that the proliferation, killing, and secretion of locally infiltrating T cells in the tumor were significantly enhanced after the combination of hypofractionation radiotherapy, and T cell depletion was reduced, indicating a comprehensive recovery of T cell function in the tumor microenvironment after the combination of hypofractionation radiotherapy and radiotherapy.

##### Differential Analysis of CD8+T Cells under Microscope

Immunofluorescence labeling of CD8+T cells was performed on the tumor tissues of each treatment group. Scanning the slices showed that under the 20× field of view, there was a significant difference in the infiltration of CD8+T cells between tumors between the treatment groups compared with the Blank group (Figure 3K). The content level of CD8+T cells stained with a red label is preliminarily visible, and from low to high, they are the Blank group, the RT (8 Gy) group, the Anti PD-1 group, the Anti PD-1 group, the RT (2 GyX4)+anti PD-1 group, and the RT (8 Gy)+anti PD-1 group.

## 4. Discussion

The treatment and immune mechanism exploration of head and neck mucosal melanoma have been the focus of clinical research in recent years. From our previous experience in the diagnosis and treatment of head and neck mucosal melanoma, it was found that existing diagnostic and treatment plans cannot effectively control the progression of head and neck mucosal melanoma, and radiotherapy combined with immunotherapy seems to be a promising clinical plan [8]. However, the specific radiotherapy mode still needs to be explored.

The value of tumor tissue α/β is generally 8–10 Gy. The value of the melanomatous α/β range is very wide, and under the LQ mode, approximately 42.5% of melanoma patients have a value of α/β ≤ 5 Gy. The mode of hypofractionation radiotherapy is more effective and with a stronger killing power for lower α/β (≤5 Gy). So, hypofractionation radiotherapy is theoretically more suitable for the treatment of melanoma patients compared with conventional radiotherapy. In addition, hypofractionation radiotherapy (>8 Gy) is more conducive to the occurrence of the “distant effect” (AE) and has a significant impact on the immune response of cytotoxic T lymphocytes (CTL), natural killer (NK) cells, and DC cells. DNA damage, oxidative stress, and cell death caused by ionizing radiation form pathogen-associated molecular patterns (PAMPs) and damage-associated molecular patterns (DAMPs). The immune system recognizes cytokines released by dead tumor cells (high mobility group protein B1 (HMGB1), heat shock proteins (HSPs), S100, oxidized DNA, ATP, etc.) [9,10] and further presents them to CTL, releasing TNF, including TNF- α And IFN- γ. When inflammatory cytokines migrate to distant tumors and metastasize, they can also activate CTL and NK cells, thus observing distant effects [11].

The combined application of immunotherapy can enhance this anti-tumor immune effect. Previous clinical studies have found that patients receiving hypofractionation radiotherapy combined with PD-1 inhibitors have increased levels of Ki67+CD8+T cells in their blood [12]. Compared with the two treatment methods used alone, hypofractionation radiotherapy (10 Gy) combined with immunosuppressive therapy is more effective in activating CD8+T levels, and the levels of Granzyme B+CD8+T cells are also significantly increased [13]. This is consistent with the conclusion of our study for Granzyme B+CD8+T cells (killer T cells), Ki67+CD8+T cells (proliferative T cells), IFN- γ+, and the levels of CD8+T cells (secretory T cells). The increase in local and distant lesions after combined therapy confirms the immunomodulatory function of hypofractionation radiotherapy combined with PD-1 inhibitors. Moreover, hypofractionation radiotherapy combined with PD-1 inhibitors can effectively inhibit the level of PD-1+CD8+T cells in CD8+T cells. There is a negative correlation between T cell function and PD-1 expression level [14]. There are also many domestic studies that have found a correlation between PD-1+CD8+T cell levels and poorer prognosis in tumor patients [15,16]. This is also consistent with the results in this study, indicating that hypofractionation radiotherapy combined with PD-1 inhibitors can improve the local depletion of CD8+T cells mediated by PD-1/PD-L1 in tumors by inhibiting the levels of PD-1+CD8+T cells. Moreover, the combination of hypofractionation radiotherapy and PD-1 inhibitors can inhibit the infiltration of Treg cells in the local tumor area, indicating the regulatory effect of this treatment method on immunosuppressive cells and further assisting in the occurrence of anti-tumor immunity. In addition, we found in the analysis of the proportion of effector T cells and initial T cells in each sample that hypofractionation radiotherapy combined with PD-1 inhibitors can increase the transformation of effector T cells in peripheral blood and secondary lymphoid organs, which is also one of the possible reasons for the systemic immune activation of this combination therapy method that we found.

Therefore, we believe that the increase in immunogenic substances in TME caused by radiation therapy leads to an increase in antigen presentation. By releasing chemokines, it increases the infiltration of CTL in TME and activates CTL cell function, inhibiting immunosuppressive cells, including Treg cells. The PD-1 upregulation factor released by tumor cells mediates the upregulation of PD-1/PD-L1. PD-1 inhibitors can prevent CTL apoptosis and maintain CTL activity by blocking PD-1, thereby achieving long-lasting anti-tumor immune function.

Previous preclinical studies have also confirmed the effectiveness of this combination therapy model. A study published in *Nature* used the mouse-derived cell line B16F10 to construct a bilateral tumor model in C57BL/6 mice. The combination of a single dose of 20 Gy irradiation and CTLA-4 antibody showed significant survival benefits (*p* < 0.001) [17]. The same conclusion was also validated in a study from 2018 on simulating distant brain metastases [18]. In that study, treatment groups with different radiation doses and anti-PD-1 antibody groups were established. From the TGI of local and distant lesions in mice, it can be seen that hypofractionation radiotherapy combined with immunosuppressive therapy has shown significant therapeutic advantages, especially for doses of 8 Gy. This indicates that hypofractionation radiotherapy can synergistically activate the systemic immune state based on PD-1 inhibitors, which is more conducive to the control of local and distant lesions. This is also consistent with previous results in the literature [19]. In addition to preclinical studies, there have also been consecutive clinical case reports indicating that some patients can have significant survival benefits and tumor relief effects from hypofractionation radiotherapy combined with immunosuppressive therapy [20,21,22]. A clinical retrospective study of radiotherapy combined with immunosuppressive agents also confirmed that this treatment mode is more conducive to tumor control compared with a single treatment regimen, but the occurrence of distant effects is not consistent [23,24,25]. A phase I clinical trial targeting 22 patients with multiple melanoma metastases was conducted using hypofractionation radiotherapy (8 GyX3) combined with anti-CTLA4 antibody ipril monoclonal antibody. The results showed that 18% of patients presented with PR, 18% with SD, and 64% with PD. The effect was not ideal. Subsequent laboratory studies by the researchers confirmed that hypofractionation radiotherapy (8 GyX3) combined with an anti-CTLA4 antibody and PD-1 inhibitor can compensate for the impact of CTLA4 antibody on CD8+T cell depletion. So, it seems that hypofractionation radiotherapy combined with PD-1 inhibitors may be a better option [17], but large-scale clinical research and exploration are still needed [26].

At present, there are no clinical trials targeting mucosal melanoma or large-scale radiotherapy combined with immunosuppressants. Our center plans to conduct a clinical trial of hypofractionation radiotherapy combined with PD-1 inhibitors for head and neck mucosal melanoma, which is still under patient recruitment.

## 5. Conclusions

For the treatment of head and neck mucosal melanoma, compared with single immunotherapy, hypofractionation radiotherapy combined with PD-1 inhibitors can more effectively inhibit tumor growth and produce distant effects by activating the systemic CD8+T cell immune response. It is expected to be promoted and validated in clinical practice, improving the cure rate and immune response rate of head and neck mucosal melanoma. Also, the immune mechanism needs further exploration.

## Figures and Tables

**Table 1:** Information on each treatment group.

Treatment Group	Treatment Plan
Anti PD-1	PD-1 inhibitor of 10 mg/kg for 2–3 weeks, BIW
RT (8 Gy)+anti PD-1	8 Gy single-dose irradiation on one side for 1 time + PD-1 inhibitor of 10 mg/kg for 2–3 weeks, BIW
RT (2 GyX4)+anti PD-1	2 Gy single-dose irradiation on one side for 4 times + PD-1 inhibitor of 10 mg/kg for 2–3 weeks, BIW
RT (8 Gy)	8 Gy single-dose irradiation on one side for 1 time + same dose of physiological saline at the same time as PD-1 inhibitor
Blank	No intervention; without irradiation + same dose of physiological saline at the same time as PD-1 inhibitor

**Table 2:** Tumor growth inhibition rates on the intervention side of each treatment group.

Treatment Group	TGI (%)			
	Day 10	Day 14	Day 18	Day 21
Anti PD-1	20.0%	29.5%	35.1%	40.4%
RT (8 Gy)+anti PD-1	58.1%	59.8%	56.2%	63.5%
RT (2 GyX4)+anti PD-1	31.8%	39.3%	35.0%	51.2%
RT (8 Gy)	11.3%	25.9%	18.8%	26.7%

Note: ≥60%, indicates strong efficacy; <60% but ≥30%, indicates moderate efficacy; <30%, indicates no efficacy.

**Table 3:** Tumor growth inhibition rates on the distant side of each treatment group.

Treatment Group	TGI (%)			
	Day 10	Day 14	Day 18	Day 21
Anti PD-1	19.3%	21.3%	20.8%	21.4%
RT (8 Gy)+anti PD-1	37.8%	42.6%	44.8%	52.0%
RT (2 GyX4)+anti PD-1	4.4%	19.0%	28.6%	30.9%
RT (8 Gy)	13.0%	14.7%	9.4%	16.9%

Note: ≥60%, indicates strong efficacy; <60% but ≥30%, indicates moderate efficacy; <30%, indicates no efficacy.

## Data Availability

Dataset available on request from the authors. The raw data supporting the conclusions of this article will be made available by the authors on request.

## References

[1] Yin G., Guo W., Chen X., Huang Z. (2019). Prognosis of endoscopic surgery and traditional open resection in mucosal melanoma of the nasal cavity and paranasal sinus. Melanoma Res..

[2] Ganti A., Raman A., Shay A., Kuhar H.N., Auger S.R., Patel T., Kuan E.C., Diaz A.Z., Batra P.S., Tajudeen B.A. (2019). Treatment modalities in sinonasal mucosal melanoma: A national cancer database analysis. Laryngoscope.

[3] Kandoth C., McLellan M.D., Vandin F., Ye K., Niu B., Lu C., Xie M., Zhang Q., McMichael J.F., Wyczalkowski M.A. (2013). Mutational landscape and signifificance across 12 major cancer types. Nature.

[4] Hamid O., Robert C., Ribas A., Hodi F.S., Walpole E., Daud A., Arance A.S., Brown E., Hoeller C., Mortier L. (2018). Antitumour activity of pembrolizumab in advanced mucosal melanoma: A post-hoc analysis of KEY-NOTE-001,002,006. Br. J. Cancer.

[5] Ribas A., Puzanov I., Dummer R., Schadendorf D., Hamid O., Robert C., Hodi F.S., Schachter J., Pavlick A.C., Lewis K.D. (2015). Pembrolizumab versus investigator-choice chemotherapy for ipilimumab-refractory melanoma (KEYNOTE-002): A randomised, controlled, phase 2 trial. Lancet Oncol..

[6] Deguchi T., Maekawa N., Konnai S., Owaki R., Hosoya K., Morishita K., Nakamura M., Okagawa T., Takeuchi H., Kim S. (2023). Enhanced Systemic Antitumour Immunity by Hypofractionated Radiotherapy and Anti-PD-L1 Therapy in Dogs with Pulmonary Metastatic Oral Malignant Melanoma. Cancers.

[7] Martinov T., Fife B.T. (2016). Fractionated radiotherapy combined with PD-1 pathway blockade promotes CD8 T cell-mediated tumor clearance for the treatment of advanced malignancies. Ann. Transl. Med..

[8] Yin G., Guo W., Liu H., Huang Z., Chen X. (2022). Efficacy of radiotherapy combined with immune checkpoint inhibitors in patients with melanoma: A systemic review and meta-analysis. Melanoma Res..

[9] Krombach J., Hennel R., Brix N., Orth M., Schoetz U., Ernst A., Schuster J., Zuchtriegel G., Reichel C.A., Bierschenk S. (2018). Priming anti-tumor immunity by radiotherapy: Dying tumor cell-derived DAMPs trigger endothelial cell activation and recruitment of myeloid cells. Oncoimmunology.

[10] McLaughlin M., Patin E.C., Pedersen M., Wilkins A., Dillon M.T., Melcher A.A., Harrington K.J. (2020). Inflammatory microenvironment remodelling by tumour cells after radiotherapy. Nat. Rev. Cancer.

[11] Yang X., Guo Y., Guo Z., Si T., Xing W., Yu W., Wang Y. (2018). Cryoablation inhibition of distant untreated tumors (abscopal effect) is immune mediated. Oncotarget.

[12] Watanabe T., Firat E., Scholber J., Gaedicke S., Heinrich C., Luo R., Ehrat N., Multhoff G., Schmitt-Graeff A., Grosu A.L. (2020). Deep abscopal response to radiotherapy and anti-PD-1 in an oligometastatic melanoma patient with unfavorable pretreatment immune signature. Cancer Immunol. Immunother..

[13] Newton J.M., Hanoteau A., Liu H.C., Gaspero A., Parikh F., Gartrell-Corrado R.D., Hart T.D., Laoui D., Van Ginderachter J.A., Dharmaraj N. (2019). Immune microenvironment modulation unmasks therapeutic benefit of radiotherapy and checkpoint inhibition. J. Immunother. Cancer.

[14] Kansy B.A., Concha-Benavente F., Srivastava R.M., Jie H.B., Shayan G., Lei Y., Moskovitz J., Moy J., Li J., Brandau S. (2017). PD-1 Status in CD8^+^ T Cells Associates with Survival and Anti-PD-1 Therapeutic Outcomes in Head and Neck Cancer. Cancer Res..

[15] Shen Y., Peng L., Zou Q., Zhao Y., Ma D. (2020). Phenotype, function and clinical significance of peripheral blood CD8^+^PD-1^+^ T cels in patients with gastric cancer. Chongqing Med..

[16] Zhou L., Ren F., Zhang G., Lu X. (2015). Relationship between the expression of PD-1 on CD8^+^ T cell and the clinical pathological parameters in non-small cell lung cancer. Chin. Clin. Oncol..

[17] Twyman-Saint Victor C., Rech A.J., Maity A., Rengan R., Pauken K.E., Stelekati E., Benci J.L., Xu B., Dada H., Odorizzi P.M. (2015). Radiation and dual checkpoint blockade activate non-redundant immune mechanisms in cancer. Nature.

[18] Pfannenstiel L.W., McNeilly C., Xiang C., Kang K., Diaz-Montero C.M., Yu J.S., Gastman B.R. (2018). Combination PD-1 blockade and irradiation of brain metastasis induces an effective abscopal effect in melanoma. Oncoimmunology.

[19] Park S.S., Dong H., Liu X., Harrington S.M., Krco C.J., Grams M.P., Mansfield A.S., Furutani K.M., Olivier K.R., Kwon E.D. (2015). PD-1 Restrains Radiotherapy-Induced Abscopal Effect. Cancer Immunol. Res..

[20] Baba K., Nomura M., Ohashi S., Hiratsuka T., Nakai Y., Saito T., Kondo Y., Fukuyama K., Kikuchi O., Yamada A. (2020). Experimental model for the irradiation-mediated abscopal effect and factors influencing this effect. Am. J. Cancer Res..

[21] Maity A., Mick R., Huang A.C., George S.M., Farwell M.D., Lukens J.N., Berman A.T., Mitchell T.C., Bauml J., Schuchter L.M. (2018). A phase I trial of pembrolizumab with hypofractionated radiotherapy in patients with metastatic solid tumours. Br. J. Cancer.

[22] Postow M.A., Callahan M.K., Barker C.A., Yamada Y., Yuan J., Kitano S., Mu Z., Rasalan T., Adamow M., Ritter E. (2012). Immunologic correlates of the abscopal effect in a patient with melanoma. N. Engl. J. Med..

[23] Dewan M.Z., Galloway A.E., Kawashima N., Dewyngaert J.K., Babb J.S., Formenti S.C., Demaria S. (2009). Fractionated but not single-dose radiotherapy induces an immune- mediated abscopal effect when combined with anti-CTLA-4 antibody. Clin. Cancer Res..

[24] Liniker E., Menzies A.M., Kong B.Y., Cooper A., Ramanujam S., Lo S., Kefford R.F., Fogarty G.B., Guminski A., Wang T.W. (2016). Activity and safety of radiotherapy with anti-PD-1 drug therapy in patients with metastatic melanoma. Oncoimmunology.

[25] Ahmed K.A., Stallworth D.G., Kim Y., Johnstone P.A., Harrison L.B., Caudell J.J., Yu H.H., Etame A.B., Weber J.S., Gibney G.T. (2016). Clinical outcomes of melanoma brain metastases treated with stereotactic radiation and anti-PD-1 therapy. Ann. Oncol..

[26] Funck-Brentano E., Baghad B., Fort M., Aouidad I., Roger A., Beauchet A., Otmezguine Y., Blom A., Longvert C., Boru B. (2020). Efficacy of late concurrent hypofractionated radiotherapy in advanced melanoma patients failing anti-PD-1 monotherapy. Int. J. Cancer.

